# Effects of miR-373 Inhibition on Glioblastoma Growth by Reducing Limk1 *In Vitro*

**DOI:** 10.1155/2020/7671502

**Published:** 2020-09-28

**Authors:** Tao Peng, Tiejun Wang, Guohui Liu, Lixiang Zhou

**Affiliations:** ^1^Department of Neurosurgery, The First Hospital of Jilin University, Xinmin Street 1#, Changchun 130021, China; ^2^Division of Orthopaedic Traumatology, The First Hospital of Jilin University, Xinmin Street 1#, Changchun 130021, China; ^3^Emergency Surgery, The First Hospital of Jilin University, Xinmin Street 1#, Changchun 130021, China

## Abstract

Glioblastoma (GBM) is an aggressive brain tumor with shorter median overall survival time. It is urgent to find novel methods to enhance the therapeutic efficiency clinically. miR-373 is related to the biological development process of cancers, but there are no reports whether modulation on miR-373 could affect GBM development or modify the efficiency of chemo- or radiotherapy yet. Our current study found that the higher level of miR-373 was observed in U-251 cells. Inhibition on miR-373 could reduce the U-251 cell number by 65% and PCNA expression obviously. In addition, inhibition on miR-373 sensitized U-251 cells to chemo- or radiotherapy. The cell cycle of U-251 cells could be modulated by miR-373 knockdown, which could enhance the p21 expression and reduce the cdc2 level. Anti-miR-373 could increase the Bax/Bcl-2 ratio of U-251 cells and induce cell apoptosis significantly. These above effects of miR-373 could be reversed by Limk1 overexpression. Thus, our experimental data confirmed the fact that miR-373 could be a new therapeutic target to enhance the efficiency of chemo- or radiotherapy for clinical GBM patients.

## 1. Introduction

Glioblastoma is an aggressive brain tumor with shorter median overall survival time in adults [1], although multiple therapeutic methods have been used including surgical resection and chemo- or radiotherapy [2, 3]. It has been reported that only a median survival of 15 months was obtained after radiotherapy combined with temozolomide (TMZ) [4, 5], which was the major chemotherapy drug for GBM but with resistance effects for most patients yet [6, 7]. Thus, it is urgent to find novel methods to enhance the therapeutic efficiency clinically. Recently, there are many studies that focused on the combination administration on tumor including the normal chemo- or radiotherapy and targeted gene treatment [8, 9], which could effectively reduce or block the resistance from antitumor therapy.

It is more important to find the novel targeted molecule by *in vitro* or *in vivo* experimental evidence, which could reduce the side effects and prolong the median survival time in the clinical therapy on GBM patients. miRNAs are endogenous, small (~22 nt) noncoding RNAs that were first reported in 1993. Its function was recognized by binding to the 3′-untranslational region (3′-UTR) of target mRNA [10–12]. Several miRNAs with an up- or downregulation level have been reported, and such abnormal expression was related to the biological development process of cancers, including cell apoptosis, cell cycle modulation, proliferation, invasion, and metastasis [13, 14]. The higher level of miR-221 [15] and lower level of miR-181 [16] were observed in clinical samples from GBM patients. Therefore, changing the level of above oncogenes or tumor suppressors by using molecular biological methods could provide great assistance to clinical therapy of tumor patients. In addition, some reports showed [17–19] that the resistance of chemo- or radiotherapy on tumor clinical treatment was also related to the effects of miRNAs, such as miR-100 [20], miR-21 [21], miR-373 [22, 23], but the underlying mechanism was still unclear.

Furthermore, it has been evidenced that miR-373 may promote migration and metastasis of cancer cells *in vitro* by regulating the CD44 [24]. HIF-1*α* could affect the miR-373 level in response to hypoxia [25]. As an oncogenic miRNA, miR-373 plays the key role in tumorigenesis through p53-mediated CDK effect [26]. But there are no reports whether modulation on miR-373 could affect GBM development or modify the efficiency of chemo- or radiotherapy on GBM. Further experimental evidence is needed both *in vitro* or *in vivo* to clarify the above underlying mechanism. In addition, the downstream effector or pathway related to cell proliferation, cell cycle, or apoptosis also should be explored or discussed in detail. Increasing evidence showed that Limk1 was a biomarker of squamous cell carcinoma [27], lung cancer [28], breast cancer [29], and even GBM [30, 31]. Limk1 could enhance the tumor cell proliferation and invasion by the MMP or p21-related pathway [32, 33]. But there is no study on the relationship between miR-373 and Limk1, especially in the pathological process of GBM.

The purpose of the current study was to investigate the roles of miR-373 in the biological behavior of GBM development. It is hypothesized that reducing miR-373 could change the proliferation or apoptosis of GBM cell *in vitro* and even sensitize GBM to chemo- or radiotherapy, which may provide the new therapeutic strategy for clinical patients.

## 2. Materials and Methods

### 2.1. Cell Culture

The human GBM cell line U-251 was purchased from the American Type Culture Collection (ATCC, Manassas, VA). Cells were cultured in DMEM (Gibco, Carlsbad, CA, USA) supplemented with 100 *μ*g/mL streptomycin, 100 U/mL penicillin, and 10% FBS, at 37°C with 5% CO_2_.

For the transfection procedure, Lipofectamine™ RNAiMAX (Thermo Fisher Scientific, IL, USA) was used as the transfection reagent [34, 35]. And anti-miR-373 (ACUCAAAAUGGGGGCGCUUUCC) and the negative control were got from the company of Thermo Fisher Scientific (IL, USA). The Limk1-mutant construct was obtained from OriGene (Beijing, China). The following experiments were performed after above transfection for 48 h.

### 2.2. Quantitative PCR

The real-time quantitative PCR was performed to detect the miR-373 and the mRNA level of its related molecule. The TaqMan assay (ABI, Forest City, CA) was used in above experiments according to the standard protocol [36].

### 2.3. Western Blot

U-251 cells were cultured and collected for western blot experiments after the following different treatments. Briefly, cells were lysed as described in previous studies [37] with the following buffer including 2 mM NaF, 0.01 M Na_3_VO_4_, 1 *μ*g/mL leupeptin, 0.01 M EDTA, 20 mM Tris-HCl, and 1% Triton X-100. Total protein was obtained after centrifugation at 12,000 rpm at 4°C for 20 min. After that, a BCA test (Pierce, Rockford, IL, USA) was used to determine the protein concentrations. Protein expression was detected by incubation with a primary antibody after the protocol of SDS-PAGE gel and transferred membranes. *β*-Actin was used to control and correct for above procedure.

### 2.4. Colony Formation Assay

U-251 cells were cultured with 6-well tissue culture plates, and the method of Giemsa stain was used to evaluate the colony formation after chemo- or radiotherapy in the presence or absence of anti-miR-373 transfection. The number of colonies was counted by light microscopy.

### 2.5. Chemo- and Radiotherapy on U-251 Cells

U-251 cells were cultured and received the following treatments. For the radiotherapy group, the *γ*-ray ionizing radiation (IR) was treated for U-251 cells by using a ^60^Co source. The dose was set at 2.4 Gy/min. For the chemotherapy group, temozolomide (TMZ, Tocris) was used at 30 nM.

### 2.6. Cell Proliferation

Cells were seeded in a 96-well plate and used to evaluate the cell proliferation by using an MTT kit (Roche Diagnostics, Germany) after chemo- or radiotherapy in the presence or absence of anti-miR-373 transfection. Following the protocol, the 10 *μ*L MTT reagent and 100 *μ*L DMSO were added, respectively. After incubation, the absorbance was recorded by a Microplate Spectrophotometer (BioTek, USA) at 490 nm. All tests were repeated for three times. As a biomarker of cell proliferation, PCNA (proliferating cell nuclear antigen) expression in U-251 cells was observed through western blot after above treatment. The primary PCNA antibody (Cell Signaling Technology, MA, USA) was used.

### 2.7. Cell Cycle Analysis

The changes on cell cycle-related proteins p21 and cdc2 were tested by the western blot method after above anti-miR-373 transfection. Primary antibodies against p21 and cdc2 (Cell Signaling Technology, MA, USA) were used to evaluate protein expression.

### 2.8. Cell Apoptosis Analysis

Firstly, cells were seeded and used to evaluate the cell apoptosis by FACS (Flow Cytometry, Becton Dickinson, CA, USA) after chemo- or radiotherapy in the presence or absence of anti-miR-373 transfection. The analysis was performed after PI and Annexin V-FITC incubation according to the protocol. In addition, western blot was performed to assess the expression of Bax and Bcl-2 after above anti-miR-373 transfection combined with chemo- or radiotherapy. The Bax and Bcl-2 primary antibodies were obtained from Cell Signaling Technology (MA, USA).

### 2.9. Statistical Analysis

Student's *t*-test and ANOVA were used to analyze the difference by using GraphPad Prism and SPSS 17.0. Data were shown as the mean ± standard error.

## 3. Results

### 3.1. Inhibition of miR-373 Reduced the U-251 Cell Proliferation

**Figure 1:**
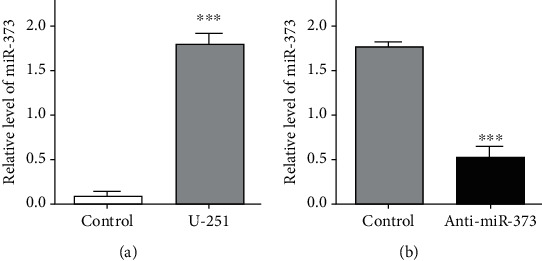
miR-373 level in U-251 cells. (a) High miR-373 level was observed by real-time quantitative PCR in U-251 cells. (b) miR-373 level was reduced obviously after anti-miR-373 transfection by real-time quantitative PCR in U-251 cells (bars indicate the standard deviation of the mean; each experiment was performed in triplicate; ^∗∗∗^*p* < 0.001).

Firstly, real-time quantitative PCR was used to investigate the miR-373 level in U-251 cells after anti-miR-373 transfection. The higher level of miR-373 was obtained in U-251 cells. It was reduced obviously by at least 71% after above transfection compared with the negative control group, which may provide the effective methods for further experiments to test the effects of miR-373 knockdown on biological changes of U-251 cells *in vitro* (Figure 1).

**Figure 2:**
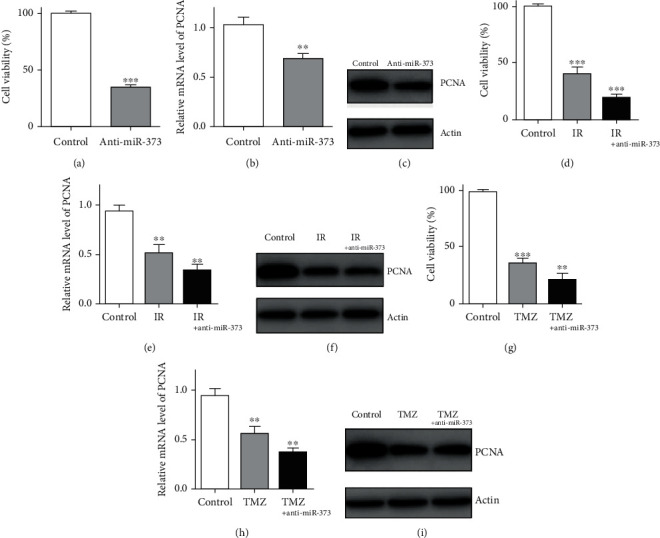
Inhibition of miR-373 reduced the U-251 cell proliferation. (a) Anti-miR-373 could reduce the U-251 cell proliferation by MTT assay. (b) PCNA mRNA level was also reduced after anti-miR-373 transfection by real-time quantitative PCR in U-251 cells. (c) Western blot results proved the lower expression of PCNA protein after anti-miR-373 transfection in U-251 cells. (d) After ionizing radiation (IR), the cell number of U-251 cells was reduced obviously by MTT assay, and additional effects were also obtained in the group of IR combined with anti-miR-373 transfection. (e) IR could also reduce the PCNA mRNA level in U-251 cells, and such inhibition was enhanced after being combined with anti-miR-373 transfection. (f) PCNA protein expression was also reduced significantly after ionizing radiation in the presence or absence of anti-miR-373 transfection. (g) After TMZ treatment, the cell number of U-251 cells was reduced obviously by MTT assay, and additional effects were also obtained in the group of TMZ treatment in the presence of anti-miR-373 transfection. (h) TMZ could also reduce the PCNA mRNA level in U-251 cells, and such inhibition was enhanced after being combined with anti-miR-373 transfection. (i) PCNA protein expression was also reduced significantly after TMZ treatment in the presence or absence of anti-miR-373 transfection (bars indicate the standard deviation of the mean; each experiment was performed in triplicate; ^∗∗∗^*p* < 0.001; ^∗∗^*p* < 0.01).

Next, MTT assay was performed to observe the effects of miR-373 inhibition on U-251 cell proliferation by anti-miR-373 transfection. Compared with the negative control group, the number of U-251 cells was decreased significantly, reduced by 65% (Figure 2).

In addition, the western blot method was used to detect the changes on PCNA expression in the presence or absence of anti-miR-373 transfection. The results showed that PCNA expression in U-251 cells was decreased obviously by reducing the miR-373 level. In addition, the results from real-time quantitative PCR proved that the lower PCNA level was obtained after above anti-miR-373 transfection (Figure 2). It has been reported that PCNA was a typical biological marker of cancer cell proliferation. Changes on its expression level after different treatments could also provide the positive evidence for effects of its regulator [38]. Thus, our results of above experiments evidenced that miR-373 knockdown has the clear inhibition effects on U-251 cell proliferation, which may help us find the novel potential therapeutic target for the treatment of clinical GBM patients.

IR resistance was the problem for such GBM patients in clinical therapy, and a novel method or molecule was needed to reduce the resistance effects. After ionizing radiation, the cell number of U-251 cells was reduced by 60% compared with the control group. After IR combined with anti-miR-373 transfection, it was decreased by 81%, which was lower than single IR-treated U-251 cells. For the PCNA expression level, both the western blot and PCR results showed that the PCNA expression level was inhibited after ionizing radiation, and such inhibition was also enhanced after being combined with anti-miR-373 transfection (Figure 2).

In the TMZ alone-treated group, the U-251 cell proliferation also decreased by 63%. And for the combined group, the number of U-251 cells was reduced at least by 79% after TMZ application in the presence of anti-miR-373 transfection. The result was clear that inhibition on the miR-373 level could enhance the therapeutic efficiency of TMZ on U-251 cells *in vitro*. For the expression of PCNA protein, it was reduced in the TMZ alone-treated group, and an obvious decrease was obtained after TMZ treatment combined with miR-373 knockdown. And a similar tendency was observed in the result of real-time quantitative PCR (Figure 2). Thus, above data also provided the experimental evidence that resistance of TMZ on GBM clinical therapy could be reversed or reduced by miR-373 knockdown, which may bring the higher therapeutic efficiency or lower toxicity for such patients.

### 3.2. Colony Formation Assay

**Figure 3:**
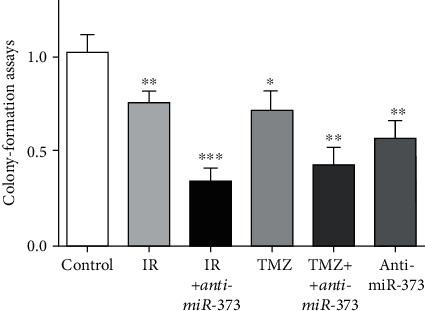
Inhibition of miR-373 reduced the U-251 cells' colony formation. Colony formation assays were performed to evaluate the effects of anti-miR-373 on U-251 cells *in vitro*. Reducing the miR-373 level, TMZ or IR treatment could decrease the colony formation of U-251 cells obviously, and additional effects were obtained in the combined groups, respectively (bars indicate the standard deviation of the mean; each experiment was performed in triplicate; ^∗∗∗^*p* < 0.001; ^∗∗^*p* < 0.01; ^∗^*p* < 0.05).

After above experiments, colony formation assay was performed to evaluate the effects of anti-miR-373 on U-251 cells *in vitro*. Reducing the miR-373 level could decrease the colony formation of U-251 cells obviously. In the TMZ or IR alone-treated group, it was reduced significantly, but additional effects were obtained in the presence of anti-miR-373, respectively (Figure 3). Our results indicated that miR-373 knockdown may reduce the colony formation capacity of U-251 cells, and such anti-miR-373 effects could sensitize U-251 cells to chemo- or radiotherapy efficiently.

### 3.3. Inhibition of miR-373 Modulated the U-251 Cell Cycle

**Figure 4:**
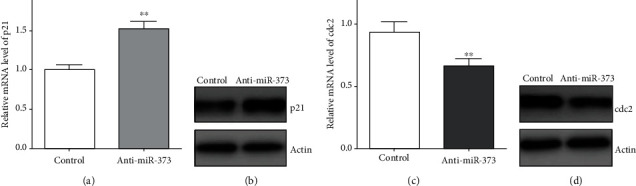
Effects of anti-miR-373 on p21 and cdc2 expression. (a) p21 mRNA level was increased in the presence of anti-miR-373 transfection by the real-time quantitative PCR, and (b) western blot result also proved that higher p21 expression was obtained after anti-miR-373 transfection; (c) cdc2 mRNA level was decreased in the presence of anti-miR-373 transfection by the real-time quantitative PCR, and (d) western blot result also proved that lower cdc2 expression was obtained after anti-miR-373 transfection (bars indicate the standard deviation of the mean; each experiment was performed in triplicate; ^∗∗^*p* < 0.01).

After reducing the miR-373 level in U-251 cells by anti-miR-373 transfection, the expression of cell cycle-related proteins such as p21 and cdc2 were observed by the western blot method. p21 expression was enhanced obviously in U-251 cells after anti-miR-373 transfection, but the expression of cdc2 was reduced. The results from the real-time quantitative PCR provided the same tendency that the higher p21 level, but lower cdc2 level, was obtained after reducing the miR-373 level in U-251 cells (Figure 4). Thus, these data demonstrated that inhibition of miR-373 cloud also modulate the U-251 cell cycle process by regulating the p21 or cdc2 level.

### 3.4. Inhibition of miR-373 Induced the U-251 Cell Apoptosis

U-251 cell apoptosis was investigated after miR-373 knockdown or combined with above treatment to evaluate the effects of miR-373 on biological changes of U-251 cells. Firstly, FACS was used to analysis the apoptotic rate of U-251 cells. In the TMZ or IR alone-treated group, the percentages of apoptotic cells were 10.9 ± 0.2% and 13.2 ± 0.6%, respectively. And it was 11.3 ± 0.5% in the anti-miR-373-transfected group. In addition, in the combined group, the percentages of apoptotic cells were 17.1 ± 0.8% and 16.4 ± 0.5% after TMZ or IR treated in the presence of anti-miR-373 transfection. Above results provided the fact that cell apoptosis could be induced by miR-373 knockdown, which also enhances the effects of chemo- or radiotherapy.

**Figure 5:**
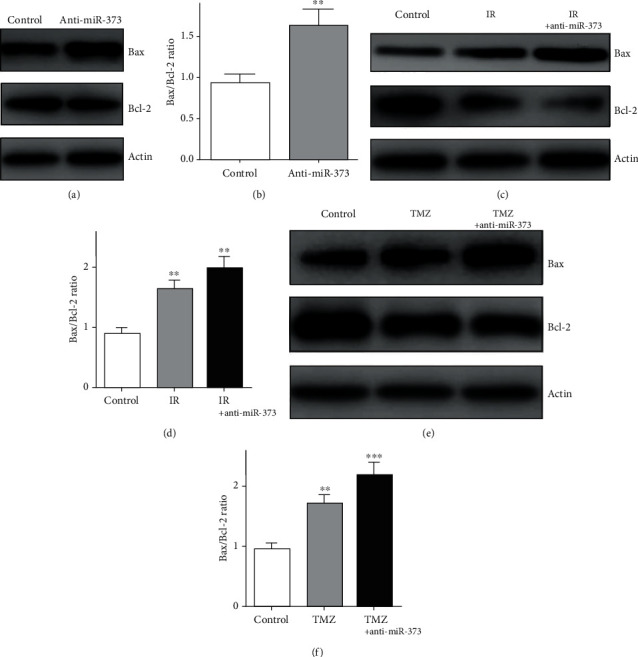
Effects of anti-miR-373 on the Bax/Bcl-2 ratio. (a, b) Anti-miR-373 could increase the Bax expression and reduce the Bcl-2 expression which resulted in the higher Bax/Bcl-2 ratio in the U-251 cells. (c, d) After ionizing radiation (IR), the Bax expression was increased combined with lower Bcl-2 expression; furthermore, the Bax/Bcl-2 ratio was also enhanced significantly after being combined with anti-miR-373 transfection. (e, f) After TMZ treatment, the Bax/Bcl-2 ratio was also increased obviously in the presence or absence of anti-miR-373 transfection, respectively (bars indicate the standard deviation of the mean; each experiment was performed in triplicate; ^∗∗∗^*p* < 0.001; ^∗∗^*p* < 0.01).

Next, the apoptosis-related proteins such as Bax and Bcl-2 were detected in U-251 cells after above treatment using the western blot method. Bax expression was increased obviously after the TMZ or IR single-treated group, which was consistent with the previous reports [39, 40]. After anti-miR-373 transfection, the western blot results showed that higher expression of Bax was obtained compared with the untreated group. In addition, in the combined group with anti-miR-373 and TMZ or IR treatment, Bax expression was enhanced significantly compared with the single-treated group (Figure 5). Thus, above data demonstrated that Bax was the downstream effector of the miR-373 molecule, which could affect the U-251 cell apoptosis.

At the same time, Bcl-2 expression was also observed after similar treatment. In the TMZ single-treated group, reduced expression of Bcl-2 was detected, and similar effects were obtained after using anti-miR-373 transfection or IR. And the Bcl-2 expression was decreased obviously in the TMZ- or IR-treated group in the presence of anti-miR-373 transfection (Figure 5).

### 3.5. Antitumor Effects of miR-373 Inhibition through Reducing Limk1 Expression

**Figure 6:**
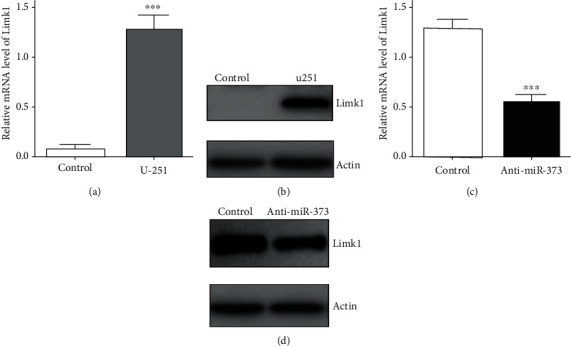
Effects of anti-miR-373 on Limk1 expression. (a) High Limk1 mRNA level was observed by the real-time quantitative PCR in U-251 cells, and (b) western blot results also proved that high Limk1 expression was obtained in U-251 cells; (c) Limk1 mRNA level was reduced by the real-time quantitative PCR in U-251 cells after anti-miR-373 transfection, and (d) lower Limk1 expression was obtained after anti-miR-373 transfection in U-251 cells (bars indicate the standard deviation of the mean; each experiment was performed in triplicate; ^∗∗∗^*p* < 0.001).

To test the relationship between miR-373 and Limk1, further experiments were investigated *in vitro*. Firstly, western blot was used to test the Limk1 expression in U-251 cells after anti-miR-373 transfection. It showed that the obvious decrease on Limk1 expression was obtained by using anti-miR-373, while higher expression of Limk1 was also observed in U-251 cells (Figure 6). In addition, further observation was performed by real-time quantitative PCR; the results showed that the Limk1 level was reduced by inhibition on miR-373 in U-251 cells (Figure 6). Thus, above results provided the direct fact that miR-373 inhibition could change the Limk1 expression in U-251 cells, which may be the downstream effector of miR-373. And the following experiments were performed to provide more details in this process.

The apoptotic rate of U-251 cells was checked again by FACS in the presence of anti-miR-373 transfection, but at the same time, Limk1 cDNA was cotransfected to test if there are different changes on the apoptotic rate in order to confirm that the above effects of anti-miR-373 might be related to its downstream effector Limk1. The results showed that the percentages of apoptotic cells were decreased after Limk1 overexpression although when using anti-miR-373 transfection, it was3.1 ± 0.2%vs.11.3 ± 0.5%, respectively, compared with the group of anti-miR-373 transfection alone. These data provided the direct information that the cell apoptosis of U-251 induced by anti-miR-373 was Limk1 dependent, which could be reversed by Limk1 overexpression. Next, chemo- or radiotherapy was combined to test the reversing effects of Limk1. After cotransfecting Limk1 cDNA, there is no difference on the apoptotic rate of U-251 cells between the chemo- or radiotherapy single-treated group and the combined group in the presence of anti-miR-373 transfection, which means sensitization on chemo- or radiotherapy by miR-373 inhibition in U-251 cells was reversed by Limk1 overexpression.

## 4. Discussion

Currently, for the treatment on GBM patients, surgery and chemo- or radiotherapy were the main available methods [2–4]. But the resistance to chemo- or radiotherapy was the trouble of the clinical treatment on these patients, which may reduce therapeutic efficacy or enhance the toxicity to normal organs by increasing the therapeutic dosage with poor long-term survival for patients [41, 42]. Temozolomide (TMZ) was the major chemotherapy drug for GBM, and surgical therapy followed with temozolomide was the standard method in the clinical treatment of glioblastoma patients currently, but most patients had the effects of temozolomide resistance that reduced the therapeutic efficiency [43, 44]. Thus, novel methods through exploring the therapeutic target to enhance therapeutic efficacy by reducing above resistance have obtained great concerns recently.

More and more studies have provided the evidence that miRNAs were related to the cell proliferation, differentiation, and apoptosis, even in development of tumors [12, 13]. Abnormal levels of miRNAs were demonstrated in the process of carcinogenesis or progression, such as lung cancer [45] and GBM [15, 16]. It has been studied that some genes such as miR-373 [22] or miR-21 [21] play an important role in above resistance effects of cancer therapy. But there were few studies on whether inhibition on miR-373 may induce above antiresistance effects in GBM treatment and which downstream effector was involved in its process, which need to be confirmed by further experimental data or evidence. Thus, the purpose of the current experiments was to test this hypothesis and to find the possible relationships between miR-373 and its downstream effector.

Our current study provided the following novelties: (1) the higher level of miR-373 was observed in U-251 cells; (2) inhibition on miR-373 could reduce the U-251 cell proliferation; (3) inhibition on miR-373 could sensitize U-251 cells to chemo- or radiotherapy; (4) the cell cycle of U-251 cells could be modulated by miR-373 knockdown; (5) inhibition on miR-373 could induce the U-251 cell apoptosis; and (6) above effects of miR-373 could be reversed by Limk1 overexpression. Thus, these experimental data confirmed the fact that miR-373 could be a new therapeutic target to enhance the efficiency of chemo- or radiotherapy for clinical GBM patients.

For cell proliferation, as a typical biological indicator [46], PCNA expression was also tested to investigate the effects of anti-miR-373 and even to observe whether combined treatment with chemo- or radiotherapy may have the additional effects. The results confirmed the above hypothesis and showed that inhibition on miR-373 may enhance the restricted effects of chemo- or radiotherapy on cell proliferation by reducing the PCNA expression.

In addition, it has been reported that Limk1 was involved in the cancer development including tumor invasion or cell apoptosis [47]. And Limk1 knockdown induced the antitumor effects for lung cancer, breast cancer, or even GBM [28–30]. As an oncogene, it was also evidenced that Limk1 was related to the resistance of chemotherapy [48, 49]. Thus, studies on the up- or downstream effector of Limk1 may provide the details of the underlying mechanism on the pathological development of GBM and even the potent novel therapeutic target for the antitumor therapy clinically.

For the apoptosis of cancer cells, the Bax/Bcl-2 ratio could provide the development on apoptosis and reflect the changes under different modulators [50]. This Bax/Bcl-2 ratio and the percentage of apoptotic cells suggested the fact that anti-miR-373 reduced the Limk1 expression, which may induce or enhance the apoptosis of U-251 cells.

Our results confirmed that inhibition on miR-373 may change the biological behavior of U-251 cells including cell proliferation, cell cycle, and even cell apoptosis. And these effects were related to its downstream effector Limk1, which could reverse above anti-miR-373 effects. Thus, reducing the miR-373 level may enhance the antiresistance effects on chemo- or radiotherapy in GBM treatment through negative regulation on Limk1. Based on above experimental evidence, miR-373 could be regarded as the predictive or prognostic biomarker for GBM patients. These two biological molecules may help us find the new strategy in the clinical setting by combining chemo- or radiotherapy to reduce the toxicity to normal tissues such as side effects and enhance the therapeutic efficiency.

## Data Availability

The data used to support the findings of this study are included within the article.
